# Structural Design and Simulation of a Multi-Channel and Dual Working Condition Wafer Defect Inspection Prototype

**DOI:** 10.3390/mi14081568

**Published:** 2023-08-07

**Authors:** Ruizhe Ding, Haiyan Luo, Zhiwei Li, Zuoda Zhou, Dingjun Qu, Wei Xiong

**Affiliations:** 1 Hefei Institutes of Physical Science, Chinese Academy of Sciences, Hefei 230031, China; drz554865890@mail.ustc.edu.cn (R.D.);; 2 Science Island Branch, Graduate School of USTC, Hefei 230026, China; 3 Key Laboratory of Optical Calibration and Characterization of Chinese Academy of Sciences, Hefei 230031, China

**Keywords:** unpattern wafer, defect inspection, dual working conditions, multi-channel, structural design

## Abstract

Detecting and classifying defects on unpatterned wafers is a key part of wafer front-end inspection. Defect inspection schemes vary depending on the type and location of the defects. In this paper, the structure of the prototype is designed to meet the requirements of wafer surface and edge defect inspection. This prototype has four inspection channels: scattering, reflection, phase, and contour, with two working conditions: surface and edge inspection. The key structure of the prototype was simulated using Ansys. The simulation results show that the maximum deformation of the optical detection subsystem is 19.5 μm and the fundamental frequency of the prototype is 96.9 Hz; thus, these results meet the requirements of optical performance stability and structural design. The experimental results show that the prototype meets the requirements of the inspection sensitivity better than 200 nm equivalent PSL spherical defects.

## 1. Introduction

Defects such as pits, scratches, and particles exist on the surface of wafers [1,2]. These defects have a significant impact on subsequent manufacturing processes and product performance. Crystal-Originated Particles (COPs) are the most typical pit defects, which can affect the integrity of the gate oxide layer, resulting in decreased device performance, increased leakage current, and even device failure [3]. The impact of scratches varies depending on their location. For instance, scratches on inter-layer dielectric (ILD) surfaces can serve as hiding places for the metal deposited in the next step, forming metal-to-metal shorts within a level [4]. During photolithography, surface particles can obstruct incident light, causing voids or shorts in integrated circuits [5,6]. These undesired occurrences modify the original circuit, resulting in device malfunction or performance degradation [7]. Crystal defects such as stacking faults and dislocations mainly affect the optical and electronic properties of devices [8]. With improvement in wafer processing technology and reduction in feature size, the harm of tiny defects to the performance of electronic components becomes increasingly severe.

In the early stage, defect inspection was mostly carried out by manual visual inspection. Due to low yield and accuracy [9], this method is gradually being replaced by Automated Defect Inspection (ADI) technology [10,11]. There are various types of wafer defect inspection technologies, which can be roughly categorized into non-optical techniques and optical techniques based on their principles. Among the commonly used non-optical techniques, KOH etching and TEM can cause damage to wafers, while SEM, CL, AFM, and MPJ have low throughput, making them unsuitable for industrial-scale production [12]. Automatic optical inspection (AOI) techniques have the advantages of non-contact, non-destructive, high throughput, and high sensitivity, and have been widely applied [13,14].

Currently, the commonly used AOI inspection channels include dark field scattering [3], bright field reflection [15,16], polarization measurement [17,18], contour measurement, and photoluminescence [19]. The dark field scattering channel can detect defects that are much smaller than the system resolution or spot size, ensuring the high sensitivity of the system to small defects [20]. Furthermore, this channel has adequate detectability of haze microroughness and crystal defects [21]. The sensitivity of the bright field reflection channel is similar to the system resolution but some defects such as macroscopic defects are clearer in this channel, and the results of this channel for macroscopic defects are more intuitive, making it a supplement to the dark field scattering channel [3]. The polarization measurement channel is based on the principle of ellipsometry and has high detection sensitivity to factors such as film thickness and stains on the surface of the wafer [22,23]. The contour measurement channel is based on the triangulation principle for distance measurement and is used to measure the height variation of the wafer surface. The photoluminescence channel is based on the fluorescence characteristics of defects in a specific band and can be used to detect sub-surface defects on the wafer [24]. In addition to the aforementioned technologies, some more advanced or targeted optical techniques have emerged recently, such as those based on terahertz (THz) waves, hyperbolic Bloch modes, or X-ray ptychography. These cutting-edge technologies are currently not mature enough but may potentially find broader applications in the future [25]. Different detection methods have their own advantages and disadvantages, and the optical characteristics of different types of defects also vary. Therefore, to improve the detection sensitivity of the instrument and increase the types of defects detected, Zhou et al. proposed to integrate multi-channel detection functions into the instrument through optical and structural design [26,27].

In addition to the detection technology, the detection area can be divided into surface and edge regions, and defects in both areas can affect product performance and yield [28,29]. Most existing instruments isolate these two working conditions; thus. different instruments need to be used for different conditions, which is cumbersome and time-consuming.

In view of the above introduction, in order to realize multiple defect inspection and integrate surface and edge detection functions, this paper studies the structure design, structure simulation, and experimental verification of multi-channel AOI technology.

This article is organized as follows: Section 2.1 briefly describes the optical detection principle of the prototype; Section 2.2 introduces the structure and implementation of the prototype; Section 2.3 carries out the static and modal simulation of the prototype structure; Section 2.4 describes the assembly and adjustment process of the prototype and shows the final product; Section 3 experimentally verifies that the structural design meets the optical performance requirements of the instrument and confirms the instrument’s capability for detecting surface defects. Section 4 gives the conclusion and summary of the paper. The results indicate that the sensitivity of the prototype is superior to 200 nm. Through multi-channel cooperation, the prototype can obtain more detailed data for subsequent processing. Through structural optimization, this design has the potential to integrate surface and edge detection functions.

## 2. Materials and Methods

### 2.1. Principles of Optics

**Figure 1:**
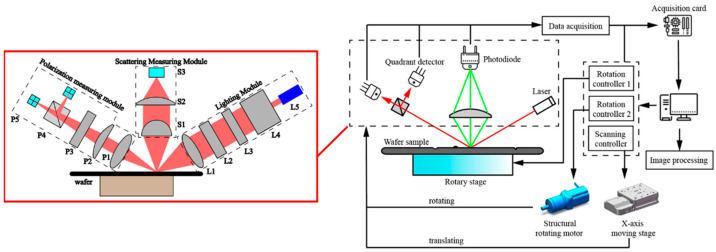
Schematic diagram of scheme principle. Lighting module: objective lens L1, half wave plate L2, polarizer L3, beam expander L4, laser (632 nm) L5; scattering measurement module: collimating lens S1, focusing lens S2, photodiode S3; polarization measurement module: collimating lens P1, deflection reset lens P2, quarter wave plate P3, polarization beam splitting cube P4, quadrant detector P5.

The prototype adopts the point scanning scheme, and the detection spot traverses the area to be measured by moving the optical machine to realize the defect inspection of the wafer surface and edge. The principle of the prototype scheme is shown in Figure 1. The upper computer controls the motion mechanism, which drives the optical machine to move according to the predetermined trajectory and then moves the detection spot. The signals at each point are converted into electrical signals by the detector and then transmitted to the upper computer via a data acquisition card for further image processing.

The optical part is divided into lighting module, scattering measurement module, and polarization measurement module according to its function. It integrates four measurement channels: scattering, reflection, phase, and contour.

In the scattering measurement channel, linearly polarized light (632 nm) is obliquely incident onto the wafer surface, and an optical system (such as a high-NA lens) is used in the normal direction to collect scattered light within a certain aperture and focus it onto a photodiode. Because the defect scatters the illumination light, there is a defect at the place where the signal is rapidly enhanced.

The illumination method for the reflection measurement channel is the same as that for the scattering measurement channel. Two quadrant detectors (QD) are arranged in the reflection direction to receive the light reflected from the substrate. The reflection signal intensity is calculated according to Equation (1) and the defect position is identified at the point where the signal rapidly decreases due to scattering.

(1) $$Specular\;signal=(A_{1}+B_{1}+C_{1}+D_{1})+k\times (A_{2}+B_{2}+C_{2}+D_{2})$$

In this equation, $A_{i},\;B_{i},\;C_{i},\;D_{i},\;i\in \{1,2\}$ represent the signal intensities received by the four pixels of quadrant detector i.

The phase measurement channel and the reflection measurement channel share a common optical path. When the illumination light (45° linearly polarized light) is reflected from the wafer surface, the amplitudes and phases of the p-polarized and s-polarized light components exhibit dissimilar changes. If Ψ and Δ represent the amplitude ratio and phase difference caused by reflection, and $I_{i},i\in \{1,2\}$ represents the light intensity of quadrant detector i, the relationship among these variables can be described by Equation (2).

(2) $$I_{1}-I_{2}=2I_{0}\left(\sin2A\sin2\Psi \cos\Delta -\cos2A\cos2\Psi \right)$$

In this equation, $I_{0}$ represents the proportionality constant of the reflected light, which is proportional to the intensity of the incident light. When the thickness of the film layer is very thin (less than 1 nm), Ψ can be considered as a constant. In this case, $I_{1}-I_{2}$ is proportional to cosΔ. Therefore, the phase difference between the two beams can be calculated based on the difference in signal intensity between the two quadrant detectors.

The contour measurement channel and the reflection measurement channel share the same optical path. Due to the tilt and height variation of the wafer surface, the spot position of the reflected beam on the quadrant detector exhibits a swinging effect. The surface profile of the wafer can be calculated based on the amount of swing [30], as shown in Equations (3) and (4):

(3) $$SCD=\frac{\left(B_{1}+C_{1}\right)-\left(A_{1}+D_{1}\right)}{A_{1}+B_{1}+C_{1}+D_{1}}$$

(4) $$SRD=\frac{\left(A_{1}+B_{1})-(C_{1}+D_{1}\right)}{A_{1}+B_{1}+C_{1}+D_{1}}$$

In the above equations, *SCD* represents the circumferential contour, which mainly reflects the height variation of the wafer surface, and *SRD* represents the radial contour, which mainly reflects the comprehensive changes in slope and height.

### 2.2. Structural Design of the Prototype

**Figure 2:**
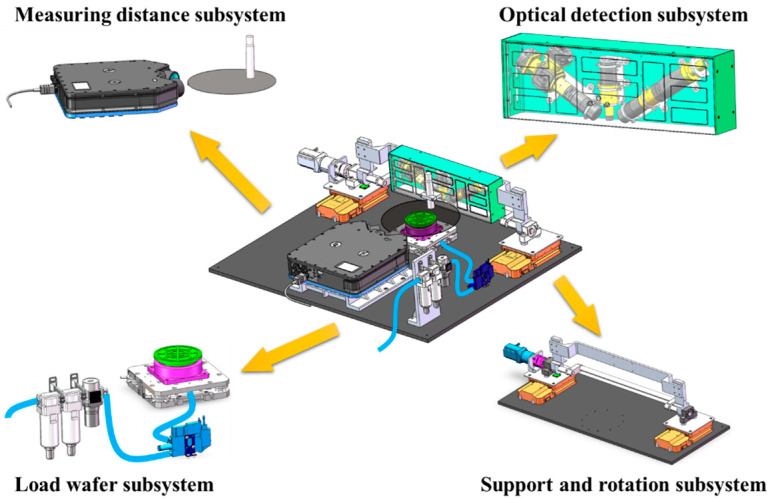
The structure of the wafer defect inspection prototype.

Based on the principles of optics, the structure of the wafer defect inspection prototype is designed as shown in Figure 2, which consists of optical detection subsystem, support and rotation subsystem, load wafer subsystem and measuring distance subsystem.

#### 2.2.1. Optical Detection Subsystem

The optical detection subsystem is modularly designed based on the schematic diagram (Figure 1), which includes the lighting module, scattering measuring module, and polarization measuring module. In accordance with the optical scheme, the following requirements are proposed for the optical detection subsystem to achieve a detection sensitivity of at least 200 nm PSL for the prototype, while ensuring that each channel works in the optimal state to leverage the advantages of multi-channel detection:

1. The diameter of the detection spot on the wafer focused by the lighting module should be less than 5 μm;
2. The focal points of the three modules should coincide, resulting in the smallest detection spot and the strongest signals from each detector;
3. In accordance with the requirements of the contour measurement channel, when the detection spot is on the wafer’s focal plane, the reflection spot should be located precisely at the center of the quadrant detectors.

**Figure 3:**
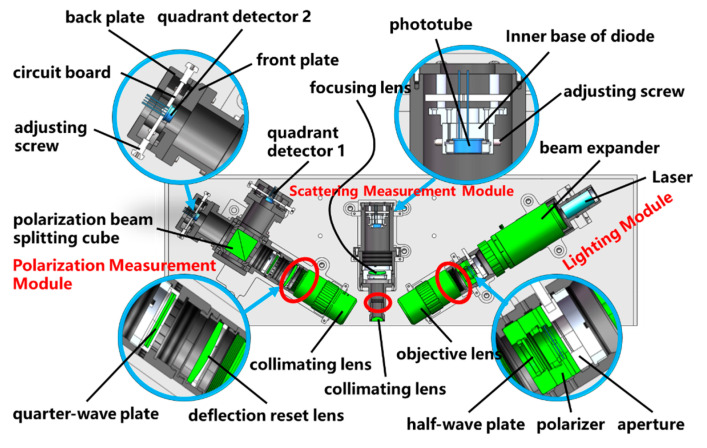
Optical detection subsystem structure diagram.

To meet the above requirements, the designed structure is shown in Figure 3. In the lighting module, a polarizer and a half-wave plate are combined to adjust the incident light to achieve the optimal polarization state. In the scattering measuring module, the collimating lens is mounted in close proximity to the front end-surface of the barrel to ensure a sufficient collection aperture angle, thereby increasing the intensity of the scattering signal and improving the sensitivity of detection. In the polarization measuring module, a fine-tuning structure is designed for the quadrant detector. The quadrant detector is mounted directly onto the circuit board, which is clamped between the front plate and the back plate. The position of the circuit board can be finely adjusted using the surrounding adjustment screws to ensure that the reflected spot is located at the center of the detector. In addition, to ensure the alignment of the focal points of each module, the objective lens and collimating lenses are all installed using threaded connections (indicated by the red circles in Figure 3), facilitating optical focusing.

#### 2.2.2. Load Wafer Subsystem

The Z-axis lifting stage is used for assisting with focusing, while the rotary stage (Akribis (Singapore), with a maximum rotational speed of 120 rpm and maximum radial and axial runout of 5 μm) is employed to drive the high-speed rotation of the wafer. The chuck is used to hold the wafer in place and the vacuum adsorption air channel, with the vacuum generating unit as the core, is utilized to provide the required vacuum pressure for wafer fixation.

The air flues 1, 2, 3, and 4 of the adsorption table are interconnected. Air flues 1 and 2 are connected to the vacuum grooves on the surface of the chuck. By replacing chucks of different sizes, wafers of varying dimensions can be securely fixed. Air flue 4 is connected to the vacuum adsorption air channel via a high-speed rotating head that is precisely located at the center of the adsorption table. This ensures the unobstructed flow of the vacuum adsorption air channel and stable fixation of the wafer during high-speed rotation. The adsorption table and rotary stage should be assembled coaxially to prevent eccentricity, which can result in missing detection, defocusing of the prototype, or damage to the rotary stage.

**Figure 4:**
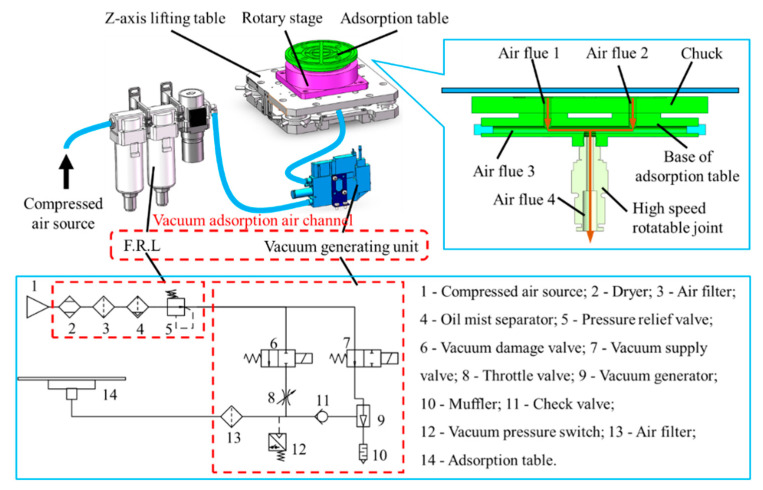
Load wafer subsystem.

To prevent damage to the wafer due to excessive impact load during the adsorption and release process, a vacuum adsorption air channel as shown in Figure 4 is designed. The F.R.L is used to filter out impurities such as particles and oil mist in the compressed air. A vacuum generator is utilized to create a vacuum environment (maximum vacuum degree is −90 KPa). The on–off control of the air channel is achieved by the vacuum supply valve and vacuum release valve (both are solenoid valves). When the vacuum supply valve is opened and the vacuum release valve is closed, the vacuum generator is connected to the compressed air source and the air inside the chuck is gradually drawn out, forming a negative pressure environment, which causes the wafer to be adsorbed. Conversely, when the vacuum release valve is opened and the vacuum supply valve is closed, the chuck is connected to the compressed air source via a throttle valve, the internal negative pressure is eliminated, and the wafer is gradually released. This smooth adsorption and release process results in reduced impact load.

Furthermore, in order to prevent wafer deformation caused by excessive vacuum pressure, it is necessary to select an appropriate vacuum degree. For a wafer with radius *R* and mass *m*, its moment of inertia *J* can be expressed as:

(5) $$J=\frac{1}{2}\cdot m\cdot R^{2}$$

The maximum angular acceleration of the rotary stage is $\alpha_{max}$; thus, the maximum torque *T* required to drive the wafer rotation is

(6) $$T=J\cdot \alpha_{\max}=\iint_{D}f_{s}\cdot \left(r\cdot d\theta \right)\cdot dr\cdot r$$

In this equation, *D* represents the adsorption surface and $f_{s}$ is the stiction of the unit adsorption surface, which can be solved according to the above equation. Therefore,

(7) $$f_{s}=\frac{\mu N}{S}=\frac{\mu \left(G+F\right)}{S}=\frac{\mu \left(G+P\cdot S^{\prime }\right)}{S}$$

In this equation, *N* represents the supporting force of the chuck, *G* is the weight of the wafer, *F* is the vacuum adsorption force, *P* is the vacuum pressure, $S^{\prime }$ is the area of the chuck’s vacuum grooves, and *S* is the area of contact between the wafer and the chuck. If the safety factor is *t*, then the required safe vacuum pressure $P_{N}$ for adsorbing the wafer is:

(8) $$P_{N}=P\cdot t=\frac{S\cdot f_{s}-\mu G}{S^{\prime }}$$

**Figure 5:**
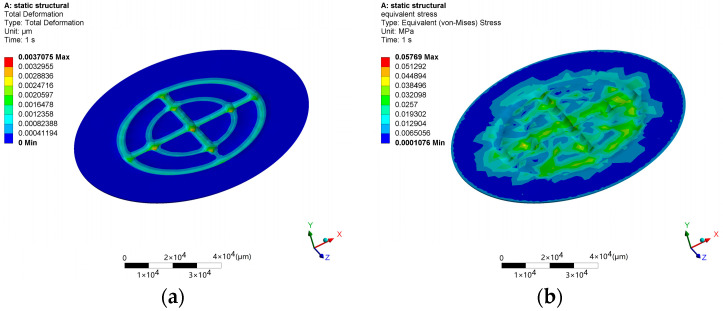
Simulation of wafer deformation. (**a**) Total deformation nephogram. (**b**) Equivalent stress nephogram.

Based on the calculations, the required vacuum degree for wafer adsorption is approximately −9.2 KPa. At this vacuum degree, simulations of wafer deformation and stress are performed based on the designed chuck, as shown in Figure 5. The results show that the maximum surface deformation of the wafer is in the nanometer range and the maximum stress is only 0.058 MPa. Therefore, this vacuum degree will not damage wafers or cause excessive deformation, and the amount of deformation is far less than the depth of focus of the optical detection subsystem, which satisfies the requirements of instrument’s optical performance.

#### 2.2.3. Support and Rotation Subsystem

**Figure 6:**
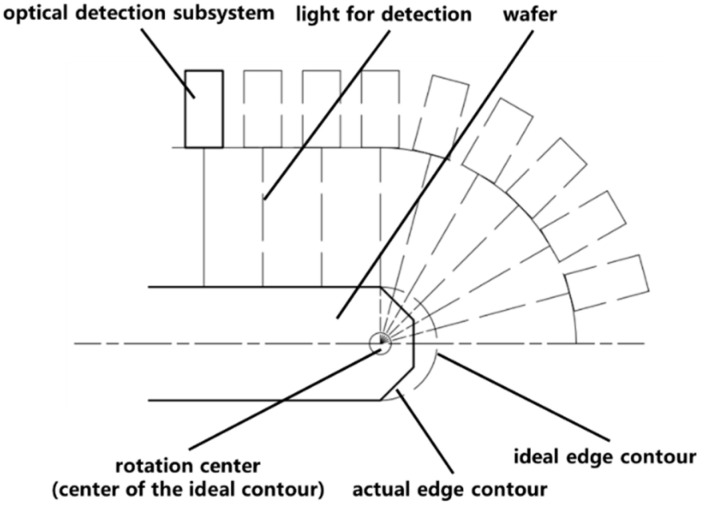
Schematic diagram of working geometry principle.

The support and rotation subsystem is the core component of the prototype for achieving dual working conditions, which is used to provide spatial motion for the optical detection subsystem. To meet the requirements of dual working conditions, the motion of the support and rotation subsystem and its accompanying optical detection subsystem should align with the working principle shown in Figure 6. For the surface defect inspection mode, the rotation axis of the rotary stage should be precisely positioned directly in front of the advancing direction of the detection light spot. As the optical detection subsystem moves radially along the wafer, the wafer should be driven to rotate at a high speed by load wafer subsystem, enabling the detection light spot to traverse the wafer surface along a spiral trajectory. For the edge defect inspection mode, for each fixed angle of rotation of the optical machine, the wafer needs to rotate a circle to complete one-cycle scan. In this mode, the situation is relatively complicated due to the fact that the ideal edge contour of the wafer is a semicircle, while the actual edge contour is irregular in shape. It is necessary to ensure that the optical detection subsystem rotates around the wafer edge, with the rotation center coinciding with the center of the ideal contour and the extension line of the light for detection passing through it, as shown in Figure 6. During the rotation process, the focal point moves along the ideal contour.

**Figure 7:**
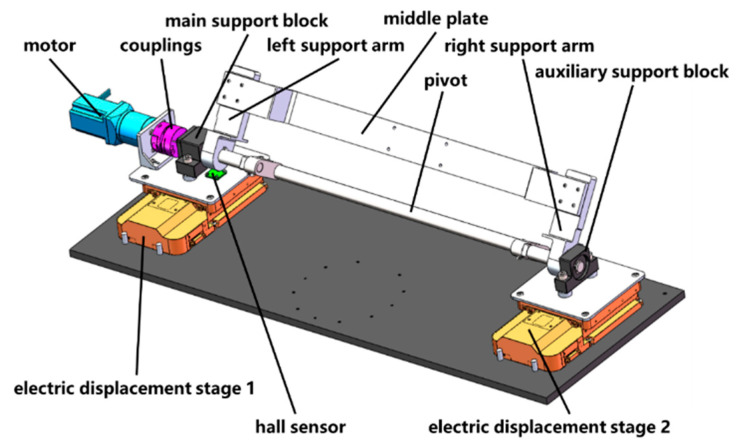
Support and rotation subsystem.

In order to realize this geometric principle, a structure as shown in Figure 7 is designed. Two parallel arranged electric displacement stages synchronize their movement through a dual-axis controller, driving the gantry and optical detection subsystem for translation. The gantry is connected to the motor via a shaft system and the torque output by the motor is transmitted to the shaft system through a coupling, thereby driving both the gantry and the optical detection subsystem rotation. The middle plate and the pivot are designed as bending structures to reserve working space for the optical detection subsystem while avoiding structural interference.

The motion accuracy and structural rigidity of the supporting rotary subsystem directly determine the detection performance of the prototype. Therefore, a reasonable selection of displacement table (travel is 50 mm, positioning accuracy is 5 μm), motor (holding torque is 50 N·m, rotor inertia is $3.6\times 10^{-2}\;kg\cdot m^{2}$), and coupling (high torque diaphragm coupling, maximum transmitted torque is 35 N·m, zero backlash) is necessary. Additionally, necessary mechanical simulations are required for this structure. The simulation details will be discussed in Section 2.3 of this paper.

#### 2.2.4. Measuring Distance Subsystem

**Figure 8:**
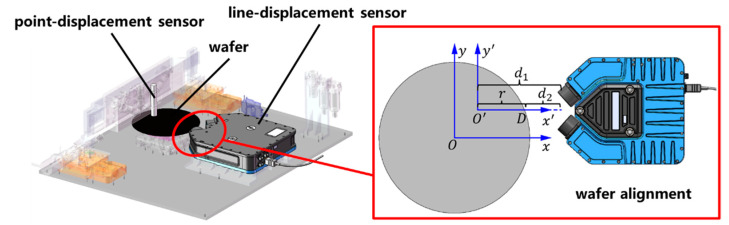
Measuring distance subsystem.

The defocus in the z-direction of the wafer and the eccentricity of the wafer symmetry axis relative to the rotary axis of the rotary stage will lead to missing detection in some areas, or reduced sensitivity of the prototype in the process of surface or edge defect inspection. To reduce defocusing and eccentricity, a measuring distance subsystem, as shown in Figure 8, has been introduced. The optical detection subsystem has been measured to have a focal depth of about 40 μm. Therefore, the defocusing and eccentricity should be limited to the micrometer level, and the corresponding detection accuracy of the detector should also be at the micrometer or sub-micrometer level.

The point-displacement sensor (self-developed in our laboratory with an accuracy of 1 μm) can obtain the distance between the detector and wafer surface, which is used to reduce defocusing. The line-displacement sensor (FocalSpec (Oulu, Finland), accuracy is 0.55 μm) can measure the distance from the wafer edge to the detector and calculate eccentricity based on the distance variation, which is used for wafer alignment [31]. After practical testing, when using the turning radius method mentioned in [31] to manually adjust the wafer position, the wafer eccentricity can be adjusted to a minimum of ±10 μm and the defocus in the z direction is limited by the stability of the Z-axis lifting stage, fluctuating within ±8 μm over 12 h, which meets the design requirements.

### 2.3. Simulation

#### 2.3.1. Static Simulation

Perform static simulation to validate the mechanical characteristics of the structure under its own weight, with a focus on the support and rotation subsystem and optical detection subsystem, which are relatively weak. Material properties are defined as shown in Table 1. Import the simplified model into Ansys and modify the contacts automatically generated by the software according to the actual situation.

**Figure 9:**
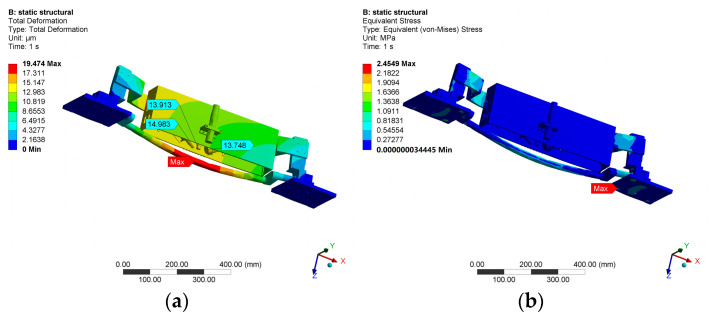
Results of static simulation. (**a**) Total deformation nephogram. (**b**) Equivalent stress nephogram.

Use Ansys to automatically mesh and then adjust locally, add self-weight, and add fixed support at the bottom of the structure. The simulation results are shown in Figure 9.

The deformation of the optical detection subsystem has the greatest impact on the performance of the prototype. According to the simulation results, the maximum deformation (15.0 μm) of the optical detection subsystem is located at the point-displacement sensor but the impact of this deformation on the performance of the prototype is relatively small. The deformation of the objective lens of the lighting module, the collimating lens of the scattering measuring module, and the collimating lens of the polarization measuring module has the greatest impact on the optical performance of the prototype. The simulation results are shown in Table 2. The Z-direction deformation can be compensated through optical focusing, while the horizontal deformation will cause the actual detection point to deviate from the theoretical detection point, affecting image reconstruction and defect localization. The simulation results show that the horizontal deformation of the aforementioned critical components is less than 10 μm, which can be solved by calibrating the prototype.

For the support and rotation subsystem, its stiffness and strength are mainly concerned. According to the simulation results, the horizontal deformation at the mechanical interface between the support and rotation subsystem and the optical detection subsystem is approximately 8.564 μm, which is a significant factor contributing to the positional deviation of the optical detection subsystem and requires optimization through structural design.

The maximum deformation (19.5 μm) of the support and rotation subsystem occur in the middle of the pivot. The overall stress on the structure is relatively small, with the maximum stress (2.4549 MPa) occurring at the bottom of the fixed plate on the right side, which is less than the allowable stress of the material and meets the design requirements.

#### 2.3.2. Modal Simulation

**Figure 10:**
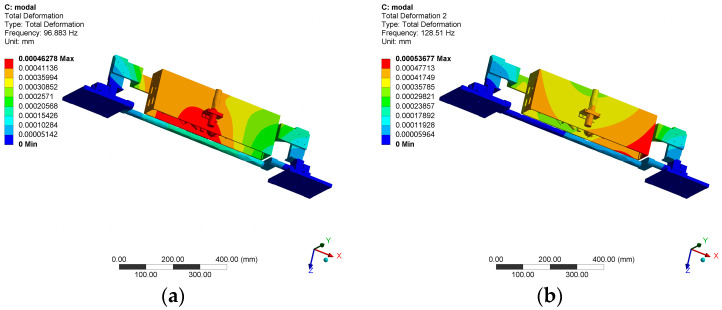
Modal vibration modes of prototype. (**a**) First-order vibration mode. (**b**) Second-order vibration mode.

To further evaluate the dynamic stiffness of the structure and prevent resonance damage, modal analysis is performed on the structure with special attention given to the relatively weak support and rotation subsystem and optical detection subsystem. The material, contact, constraint, and mesh settings are consistent with those used in static simulation. The damping effect is ignored during the analysis, and the first ten modal shapes and natural frequencies under the no pre-stress state are obtained. The vibration modes of the structure are shown in Figure 10, and the natural frequencies of each mode are listed in Table 3.

The simulation results show that the fundamental frequency of the structure is 96.883 Hz, which meets the design requirements, and the structure has good dynamic stiffness.

### 2.4. Prototype Development

**Figure 11:**
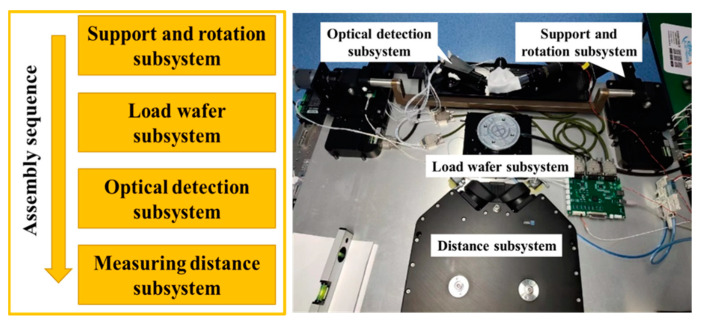
Assembly and adjustment steps and results of the prototype.

The assembly and adjustment process determines the core indicators such as detection accuracy and sensitivity of the entire machine. Assemble and adjust the prototype according to the process shown in Figure 11. The key is the assembly and adjustment of each moving axis and optical detection subsystem.

The assembly and adjustment of the moving axis are mainly to adjust the relative positions of the electric displacement stages, z-axis lifting stage, and rotary stage to ensure the accuracy of defect positioning. The assembly and adjustment of the optical detection subsystem are mainly to adjust the relative position of its internal modules. In these steps, laser interferometer, theodolite, dial indicator, etc. are mainly used. The assembly and adjustment results are shown in the right side of Figure 11.

## 3. Results

**Figure 12:**
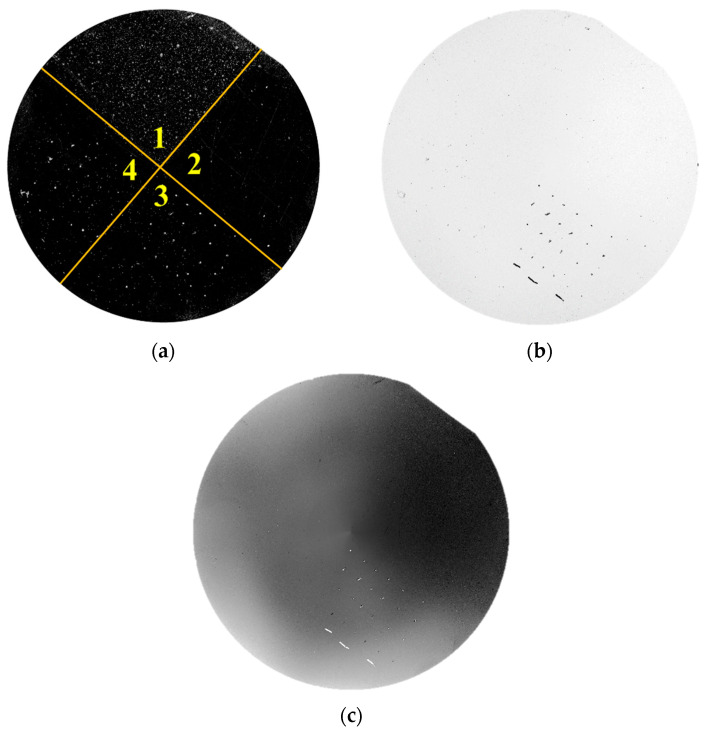
Detection results of the wafer with defects. (**a**) Result of the scatter measurement channel. (**b**) Result of the reflect measurement channel. (**c**) Result of the phase measurement channel.

Use the prototype shown in Figure 11 to detect 4-inch bare silicon wafers with defects, analyze the defect inspection ability of the prototype, and then verify the rationality of the prototype structure. As shown in Figure 12a, particles (200 nm PSL), scratches, stains, and pits were, respectively, made in areas 1–4 of the clean wafer. Due to the fact that the cleanliness level of the prototype’s working environment is only Class 10,000, in addition to the aforementioned artificially created defects, there are inevitably some large-sized particles (such as dust) uniformly distributed on the wafer surface. Use the prototype to detect the defect samples; the detection results are shown in Figure 12.

Experimental results demonstrate that the dark field scattering channel has the highest detection sensitivity. In addition to being able to clearly detect large-sized particles, it can also detect small defects such as 200 nm PSL particles, scratches, and pits. However, its detection sensitivity for stains is relatively poor. In contrast, the reflection measurement channel is better at detecting macroscopic defects such as large-sized particles, pits, and some scratches. However, it has a high sensitivity for the detection of stains, and this may be related to the characteristic of stains. The phase measurement channel has the highest sensitivity for the detection of stains but it can hardly detect any other types of defects. Therefore, to achieve accurate defect localization and classification, it is necessary to comprehensively analyze the above channel results. As previously shown, for 200 nm PSL spheres, the peak signal-to-noise ratio (PSNR) of the prototype can reach 32.07 dB, indicating its potential to detect even smaller defects [32].

**Figure 13:**
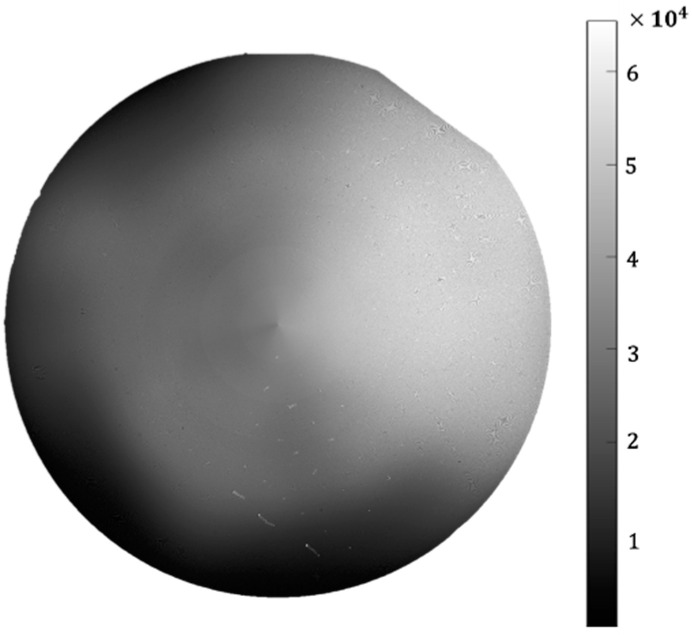
Detection results of the contour measurement channel.

The contour measurement channel mainly reflects the height fluctuation of the surface and the result is shown in Figure 13. The height of each position is positively correlated with the gray-level of this position. The height change may be caused by vacuum adsorption, the tilt of the chuck, machining error, wafer deformation, or other factors. The current test results can only reflect the relative trend of wafer surface height fluctuation. To convert it to actual height, further calibration of this channel is required. Moreover, in Figure 12c and Figure 13, undesired radial artifacts are observed, primarily due to the lack of temperature control equipment in the instrument leading to laser instability. Additionally, environmental lighting and insufficient chuck machining precision can also contribute to this issue. Therefore, in order to address this issue, we require a high-precision chuck, a temperature control device, and a light-shielding shell.

## 4. Discussion

In this work, we propose a design scheme for a dual-condition, multi-channel unpatterned wafer defect inspection prototype, which includes four measurement channels: scattering, reflection, phase, and contour. The prototype integrates surface and edge defect inspection functions. The goal of this work is to provide a prototype design scheme with high integration, low cost, and the ability to detect multiple types of defects for the defect inspection field. The article focuses on the core of the multi-channel and dual-condition detection scheme and the structure implementation of the prototype, with emphasis on the latter.

The feasibility of the structure is further verified through simulation and experimentation. The simulation shows that the structure satisfies the optical principles and mechanical requirements. In the experiment, the prototype detected defects such as particles (200 nm PSL), pits, scratches, and stains on our homemade samples, further demonstrating the rationality of the prototype’s structural design and its applicability to actual wafer defect inspection.

In summary, our prototype features four channels (scattering, reflection, phase, and contour) vs. the single-channel or dual-channel instruments proposed by Hangyeong Oh [33], Jules Karangwa [34], and Fan Wu [18]. Thereby, this prototype has the ability to acquire more detailed defect information. This information aids in preventing defect omission and improving the accuracy of defect localization, sizing, and classification. Moreover, through structural innovations, our prototype has dual working condition vs. the single working condition instruments commonly used at present like the KLA-SP series. This simplifies production processes and improves the efficiency of operators. Moreover, compared to commercial defect detection instruments under single working conditions, such as the KLA-SP series, our prototype has the potential to integrate surface and edge defect detection capabilities through structural innovations. This simplifies production processes and improves the efficiency of operators.

However, for edge defect inspection, due to the lack of autofocus function in the prototype design, the optical system loses focus during the rotation process, resulting in only about 1/3 of the edge being detected. It is difficult to achieve defect inspection for the entire edge, so further research is needed.

Therefore, regarding the prototype, the following tasks need to be conducted in the future:

1. Explore and optimize the detection limit of the prototype. Based on optical simulations, the sensitivity of the prototype can reach 100 nm. Currently, we have reached measurements up to 200 nm due to the lack of 100 nm defect samples. Further exploration and optimization will be carried out.
2. To mitigate potential defocusing issues during the detection process, an autofocus mechanism will be incorporated.
3. Prototype calibration will be conducted to mitigate the impact of factors such as machining errors, assembly errors, and structural deformations on the detection accuracy. Standard calibration boards and corresponding calibration algorithms will be employed for this purpose.
4. The prototype is currently capable of detecting defects. However, in order to apply it to practical production and fully utilize the advantages of multi-channel capability, further analysis of the multi-channel detection results is necessary. This includes defect localization, classification, sizing, and other aspects.
5. Since this prototype is only used for theoretical validation, both the rotational speed of the rotary stage and the travel range of the displacement stage are relatively small. Therefore, the current prototype is only used for the detection of 4-inch wafers and its yield is limited to 1.5 WPH. If it is used for production purposes, a high-speed (e.g., 5000 rpm) rotary stage should be selected to enhance productivity and a displacement stage with a larger travel range (e.g., 400 mm) should be chosen to detect larger wafers. Furthermore, data transmission should also be considered.

## Figures and Tables

**Table 1:** Material definition ^1^.

Material Type	$\mathrm{Density}/(\mathrm{kg}\cdot m^{-3})$	Elastic Modulus/(GPa)	Poisson’s Ratio
2A12	2770	72.4	0.33
6061	2713	69.04	0.33
Stainless steel	7750	193	0.31

^1^ All data are sourced from the material library of SolidWorks.

**Table 2:** Deformation of key parts in optical detection subsystem.

	Horizontal Deformation/(μm)			Vertical Deformation (z Direction)/(μm)	Total Deformation/(μm)
	X	Y	Total		
Objective	−1.155	−6.687	6.786	11.975	13.748
Collimating lens (Scattering measuring module)	−1.807	−7.969	8.171	11.286	13.913
Collimating lens (Polarization measuring module)	−1.903	−9.116	9.313	11.765	14.983

**Table 3:** Results of modal simulation of prototype.

Mode	Natural Frequency/(Hz)	The Position of Maximum Deformation (Excluding the Shell)
1	96.883	Objective lens of lighting module
2	128.51	Point-displacement sensor
3	152.5	Pivot
4	166.08	Point-displacement sensor
5	183.25	Point-displacement sensor
6	191.69	Objective lens of lighting module
7	227.2	Back plate of the quadrant detection component
8	262.77	Pivot
9	301.47	Objective lens of lighting module
10	353.86	Objective lens of lighting module

## Data Availability

Not applicable.
